# Synthesis and Biological Activities of 4-Aminoantipyrine Derivatives Derived from Betti-Type Reaction

**DOI:** 10.1155/2014/639392

**Published:** 2014-03-04

**Authors:** Ipsita Mohanram, Jyotsna Meshram

**Affiliations:** ^1^Department of Chemistry, Rashtrasant Tukadoji Maharaj Nagpur University, Nagpur 440033, India; ^2^Department of Organic Chemistry, School of Chemical Sciences, North Maharashtra University, Jalgaon 425001, India

## Abstract

The present work deals with the synthesis and evaluation of biological activities of 4-aminoantipyrine derivatives derived from a three-component Betti reaction. The synthesis was initiated by the condensation of aromatic aldehyde, 4-aminoantipyrine, and 8-hydroxyquinoline in presence of fluorite as catalyst in a simple one-step protocol. The reactions were stirred at room temperature for 10–15 min achieving 92–95% yield. The structures of synthesized derivatives were established on the basis of spectroscopic and elemental analysis. All derivatives **4(a–h)** were screened *in vivo* and *in vitro* for anti-inflammatory and anthelmintic activity against a reference drug, Diclofenac and Albendazole, respectively. The screening results show that compounds **4c**, **4d**, **4f**, and **4h** were found to possess potential anti-inflammatory activity while compounds **4a**, **4b**, **4e**, and **4g** are potent anthelmintic agents when compared with reference drugs, respectively. The bioactivity of these derivatives has also been evaluated with respect to Lipinski's rule of five using molinspiration cheminformatics software.

## 1. Introduction

Multicomponent reactions (MCRs) have appeared as an imperative means for the construction of diverse and complex organic molecules [1]. They have intrinsic advantages over two component reactions in several aspects including the simplicity of a one-pot procedures and possible structural variation. The synthetic competence comes from several tandem bond formation reactions in MCRs, which save time, energy, and raw material. Betti reaction is a modified type of Mannich reaction [2] which has subsequently become vital in synthetic chemistry because of C–C bond formation under mild experimental conditions. Interest in the chemistry of Betti reaction derivatives was also strengthened as it was found to possess various catalytic and biological applications [3–5]. Nonsteroidal anti-inflammatory drugs (NSAIDs) are the most clinically important medicine used for the treatment of inflammation-related diseases like arthritis, asthma, and cardiovascular diseases [6]. However, the long-term administration of NSAID may induce gastrointestinal ulcers, bleeding, and renal disorders due to their nonselective inhibition of both constitutive (COX-1) and inducible (COX-2) isoforms of the cyclooxygenase enzymes [7–9]. Therefore, new anti-inflammatory drugs lacking those effects are being searched all over the world as alternatives to NSAIDs [10]. Due to the emerging need of improved and highly selective inhibitors of COX-2, various heterocyclic compounds are synthesized amongst pyrazole compounds and their derivatives are some of them. 4-Aminoantipyrine is known for the variety of its clinical applications such as anti-inflammatory, analgesic, antipyretic [11, 12], and several chemotherapeutic agents [13]. It is evident from the reported literatures that compounds possessing pyrazole nuclei showed significant anthelmintic as well as antimicrobial activities [14–16].

Structural variations produce new physical and biological properties. The molecular manipulation of a promising lead compound is still a major line of approach for the discovery of new drugs. Molecular rearrangement involves the efforts to combine separate groups having similar activity in one compound by eliminating or substituting new moiety to a parent lead compound. Hence, an attempt has been made in this study to condense 4-aminoantipyrine in a Betti reaction to formulate novel biologically potent moieties using fluorite [17–19] as an excellent catalyst. Fluorite (also called fluorspar) is a natural occurring mineral composed of calcium fluoride (CaF_2_). It may occur as transparent or translucent isometric cubic and octahedral crystals. Fluorite acts as a mild acid in the dehydration reaction and increases the reaction rate without affecting the yield of desired products.

The paper deals with the synthesis of 4-aminoantipyrine derivatives via three-component Betti reaction and its assessment for biological applications, namely, anti-inflammatory and anthelmintic. We have also investigated the biological applications of these derivatives using online cheminformatics molinspiration software. A comparison between experimental and theoretical predictions of the biological activity has enabled us to identify alternative combined pharmacophore sites structures. The main interesting task of this work is to develop robust prediction models for inhibitory properties (solubility, bioavailability, etc.) to interpret the calculated/predicted results for the design of specific new compounds.

## 2. Materials and Methods 

### 2.1. General

All the reagents and solvents are of analytical grade purchased from a commercial source and used directly. Fluorite was purchased in the form of crystalline block from an Indian supplier and hammered into pieces of 1–3 mm in size before use. All melting points were determined by open tube capillaries method and are uncorrected. The purity of compounds was checked routinely by TLC (0.5 mm thickness) using silica gel-G coated Al-plates (Merck) and spots were visualized by exposing the dry plates in iodine vapours. IR spectra (*υ*_max⁡_ in cm^−1^) were recorded on a Schimadzu-IR Prestige 21 spectrometer using KBr technique; ^1^H NMR spectra and ^13^C NMR spectra of the synthesized compounds were recorded on a Bruker-Avance II 400 (400 MHz) and Varian-Gemini (100 MHz) spectrometer using DMSO-d_6_ solvent and TMS as an internal standard. Mass spectra were recorded on a Micromass Q-T of high resolution mass spectrometer. The elemental analysis (C, H, N, and S) of compounds was performed on Carlo Erba-1108 elemental analyzer. All the experimental protocols were approved by the Institutional Animal Ethics Committee (IAEC) of Sharad Pawar College of Pharmacy, Nagpur, India (approval number: SPCP/2013/595). The experiments and the care of the laboratory animals were according to current ethical guidelines by the Committee for the Purpose of Control and Supervision on Experiments on Animals (CPCSEA), Ministry of Environment and Forests, Government of India, New Delhi.

### 2.2. Protocol for *In Vivo* Anti-Inflammatory Assessment

Wistar albino rats [20] were divided into ten groups of six animals each. Diclofenac was used as a reference drug at 10 mg/kg and all the synthesized compounds were administered at 150 mg/kg of body weight. After one hour of the oral administration of synthesized drugs and standard drug, freshly prepared 0.1 mL carrageenin (1% carrageenin in 0.9% NaCl) was injected into the left hind limb of each rat under the subplantar aponeurosis. Measurement of paw volume was done by means of volume displacement technique using Plethysmometer [21]. Paw volume was recorded at the interval of 0, 1, 2, 3, and 4 h after carrageenin injection. Results were expressed as an increase in paw volume in comparison with the control group. Control group was administered with normal saline water. The results were expressed as mean ± S.E.M and data were statistically analyzed by one-way analysis of variance (ANOVA) and *P* < 0.05 was considered as significant.

### 2.3. Protocol for *In Vitro* Anthelmintic Investigation

Indian earthworms of the genus and species *Pheretima posthuma* [22] (family: Megascolecidae) were used for this study. The earthworms that are 3–5 cm in length and 0.1-0.2 cm in width were used for all experimental protocols. The worms were divided into the ten groups containing six earthworms in each group. Albendazole solution was used as a standard drug and saline water as control. The test compounds **4(a–h)** and Albendazole were dissolved in minimum quantity of 2% dimethyl sulfoxide (DMSO) and the volume was adjusted to 10 mL with saline water for making the concentration of 12.5, 25, 50, 100, and 150 mg/mL. The anthelmintic activity was determined in six observations. The earthworms were observed for their spontaneous motility an evoked responses. Observations were made for time taken to paralysis and death of individual worms. Paralysis was said to occur when the worms do not revive even in saline water. Death was concluded when the worms lost their motility followed by fading away of their body color. The results were expressed as mean ± S.E.M and data were statistically analyzed by one-way analysis of variance (ANOVA) and *P* < 0.05 was considered as significant.

### 2.4. Protocol for the Synthesis of 4-Aminoantipyrine Derivatives **4(a**–**h)**

**Scheme 1:**
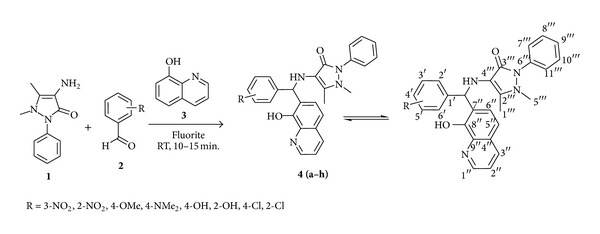
Synthesis of 4-aminoantipyrine derivatives via Betti reaction.

A mixture of 4-aminoantipyrine (0.01 mol) **1**, substituted aromatic aldehyde (0.01 mol) **2,** and 8-hydroxyquinoline (0.01 mol) **3 **was dissolved in 10 mL of 95% ethanol in one pot and was magnetically stirred at room temperature in presence of fluorite (2% weight with respect to all reactants) (Scheme 1). The reaction mixture was stirred for 10–15 min. The completion of the reaction was monitored by TLC by using mixture of ethyl acetate and hexane as mobile phase. After completion, the reaction mixture was poured into crushed ice. The crude product and catalyst were collected on a Buchner funnel by filtration. The crude product was purified by recrystallization from hot ethanol to get the pure product. The following are the spectral data of the synthesized compounds.

#### 2.4.1. 4-((3-Nitrophenyl)(8-hydroxyquinolinyl)methylamino)-1,5-dimethyl-2-phenylpyrazol-3-one (**4a**)

Yield: 95%; IR (KBr, cm^−1^): 3420 (–OH), 3325 (–NH), 3010 (Ar–H), 2970 (–CH_3_), 2872 (–CH), 1695 (C=O), 1591 (C=N), 1548 (–NO_2_). ^1^H NMR (DMSO-d_6_, ppm): 2.54 (s, 3H, –CH_3_), 2.83 (s, 1H, –NH), 3.26 (s, 3H, –CH_3_), 5.33 (s, 1H, –OH), 5.45 (s, 1H, –CH), 6.71–7.14 (m, 5H, Ar), 7.18–7.40 (m, 4H, Ar), 7.45–8.21 (m, 5H, Ar). ^13^C NMR (DMSO-d_6_, ppm): 144.4 (C1′), 124.5 (C2′), 149.9 (C3′), 120.4 (C4′), 131.2 (C5′), 134.7 (C6′), 153.2 (C1′′), 126.4 (C2′′), 135.6 (C3′′), 127.5 (C4′′), 124.7 (C5′′), 129.6 (C6′′), 122.4 (C7′′), 148.2 (C8′′), 136.8 (C9′′), 53.0 (–CH), 128.5 (C1′′′), 135.6 (C2′′′), 161.6 (C3′′′), 117.4 (C4′′′), 33.8 (C5′′′), 133.5 (C6′′′), 120.6 (C7′′′), 127.4 (C8′′′), 121.2 (C9′′′), 127.9 (C10′′′), 120.9 (C11′′′). MS (C_27_H_23_N_5_O_4_): *m/z* 481.18 (M^+^, 100%). Elemental analysis: calcd. (found): C, 71.35 (71.30); H, 4.77(4.76); N, 15.12 (15.09).

#### 2.4.2. 4-((2-Nitrophenyl)(8-hydroxyquinolinyl)methylamino)-1,5-dimethyl-2-phenylpyrazol-3-one (**4b**)

Yield: 88%; IR (KBr, cm^−1^): 3422 (–OH), 3328 (–NH str.), 3025 (Ar–H), 2972 (–CH_3_), 2874 (–CH), 1690 (C=O), 1590 (C=N), 1543 (–NO_2_). ^1^H NMR (DMSO-d_6_, ppm): 2.53 (s, 3H, –CH_3_), 2.81 (s, 1H, –NH), 3.21 (s, 3H, –CH_3_), 5.31 (s, 1H, –OH), 5.42 (s, 1H, –CH), 6.74–7.16 (m, 5H, Ar), 7.20–7.33 (m, 4H, Ar), 7.42–8.22 (m, 5H, Ar). ^13^C NMR (DMSO-d_6_, ppm): 143.1 (C1′), 124.7 (C2′), 150.1 (C3′), 120.3 (C4′), 131.0 (C5′), 134.5 (C6′), 151.6 (C1′′), 126.8 (C2′′), 134.7 (C3′′), 127.1 (C4′′), 124.5 (C5′′), 129.9 (C6′′), 121.9 (C7′′), 148.7 (C8′′), 135.5 (C9′′), 47.6 (–CH), 128.1 (C1′′′), 135.5 (C2′′′), 161.7 (C3′′′), 117.2 (C4′′′), 33.2 (C5′′′), 133.5 (C6′′′), 120.5 (C7′′′), 127.5 (C8′′′), 120.5 (C9′′′), 127.7 (C10′′′), 120.3 (C11′′′). MS (C_27_H_23_N_5_O_4_): *m/z* 481.18 (M^+^, 100%). Elemental analysis: calcd. (found): C, 73.53 (73.56); H, 4.87(4.86); N, 15.10 (15.12).

#### 2.4.3. 4-((4-Methoxyphenyl)(8-hydroxyquinolinyl)methylamino)-1,5-dimethyl-2-phenylpyrazol-3-one (**4c**)

Yield: 92%; IR (KBr, cm^−1^): 3426 (–OH), 3327 (–NH), 3028 (Ar–H), 2971 (–CH_3_), 2870 (–CH), 1693 (C=O), 1593 (C=N), 1247 (–OCH_3_). ^1^H NMR (DMSO-d_6_, ppm): 2.51 (s, 3H, –CH_3_), 2.82 (s, 1H, –NH), 3.23 (s, 3H, –CH_3_), 3.73 (s, 3H, –OCH_3_), 5.35 (s, 1H, –OH), 5.41 (s, 1H, –CH), 6.72–6.81 (m, 5H, Ar), 6.95–7.18 (d, 4H, Ar), 7.26–8.18 (m, 5H, Ar). ^13^C NMR (DMSO-d_6_, ppm): 143.3 (C1′), 124.5 (C2′), 151.2 (C3′), 120.1 (C4′), 131.5 (C5′), 134.6 (C6′), 150.1 (C1′′), 125.5 (C2′′), 135.5 (C3′′), 126.6 (C4′′), 124.3 (C5′′), 121.3 (C6′′), 121.7 (C7′′), 147.7 (C8′′), 135.3 (C9′′), 52.1 (–CH), 128.5 (C1′′′), 135.3 (C2′′′), 161.0 (C3′′′), 116.8 (C4′′′), 33.6 (C5′′′), 132.5 (C6′′′), 121.2 (C7′′′), 126.4 (C8′′′), 121.2 (C9′′′), 128.1 (C10′′′), 121.4 (C11′′′). MS (C_28_H_26_N_4_O_3_): *m/z* 466 (M^+^, 100%). Elemental analysis: calcd. (found): C, 70.03 (70.05); H, 5.71(5.76); N, 13.90 (13.92).

#### 2.4.4. 4-((4-(Dimethylamino)phenyl)(8-hydroxyquinolinyl)methylamino)-1,5-dimethyl-2-phenylpyrazol-3-one (**4d**)

Yield: 83%; IR (KBr, cm^−1^): 3428 (–OH), 3334 (–NH), 3015 (Ar–H), 2973 (–CH_3_), 2872 (–CH), 1695 (C=O), 1591 (C=N), 1581 (–CN). ^1^H NMR (DMSO-d_6_, ppm): 2.52 (s, 3H, –CH_3_), 2.84 (s, 1H, –NH), 2.88 (s, 6H, –N(CH_3_)_2_), 3.25 (s, 3H, –CH_3_), 5.32 (s, 1H, –OH), 5.43 (s, 1H, –CH), 6.73–6.88 (m, 5H, Ar), 6.98–7.20 (d, 4H, Ar), 7.25–8.23 (m, 5H, Ar). ^13^C NMR (DMSO-d_6_, ppm): 144.6 (C1′), 125.0 (C2′), 149.7 (C3′), 119.3 (C4′), 131.3 (C5′), 134.5 (C6′), 153.4 (C1′′), 126.4 (C2′′), 135.6 (C3′′), 126.8 (C4′′), 124.9 (C5′′), 128.7 (C6′′), 122.3 (C7′′), 148.3 (C8′′), 136.7 (C9′′), 53.0 (–CH), 128.7 (C1′′′), 135.8 (C2′′′), 161.2 (C3′′′), 116.7 (C4′′′), 33.4 (C5′′′), 132.3 (C6′′′), 120.3 (C7′′′), 126.6 (C8′′′), 121.5 (C9′′′), 128.3 (C10′′′), 120.6 (C11′′′). MS (C_29_H_29_N_5_O_2_): *m/z* 479 (M^+^, 100%). Elemental analysis: calcd. (found): C, 73.53 (73.56); H, 4.77(4.76); N, 13.01 (13.05).

#### 2.4.5. 4-((4-Hydroxyphenyl)(8-hydroxyquinolinyl)methylamino)-1,5-dimethyl-2-phenylpyrazol-3-one (**4e**)

Yield: 91%; IR (KBr, cm^−1^): 3423 (–OH), 3332 (–NH), 3020 (Ar–H), 2970 (–CH_3_), 2871 (–CH), 1690 (C=O), 1590 (C=N). ^1^H NMR (DMSO-d_6_, ppm): 2.53 (s, 3H, –CH_3_), 2.81 (s, 1H, –NH), 3.24 (s, 3H, –CH_3_), 5.08 (s, 1H, –OH), 5.31 (s, 1H, –OH), 5.44 (s, 1H, –CH), 6.71–6.84 (m, 5H, Ar), 6.98–7.16 (d, 4H, Ar), 7.27–8.18 (m, 5H, Ar). ^13^C NMR (DMSO-d_6_, ppm): 144.4 (C1′), 124.4 (C2′), 149.8 (C3′), 119.2 (C4′), 130.6 (C5′), 134.7 (C6′), 152.5 (C1′′), 125.3 (C2′′), 134.9 (C3′′), 127.4 (C4′′), 124.4 (C5′′), 128.6 (C6′′), 122.4 (C7′′), 148.2 (C8′′), 135.9 (C9′′), 53.0 (–CH), 128.9 (C1′′′), 135.3 (C2′′′), 161.6 (C3′′′), 117.1 (C4′′′), 33.1 (C5′′′), 133.3 (C6′′′), 120.6 (C7′′′), 127.2 (C8′′′), 120.3 (C9′′′), 127.5 (C10′′′), 120.8 (C11′′′). MS (C_27_H_24_N_4_O_3_): *m/z* 452 (M^+^, 100%). Elemental analysis: calcd. (found): C, 71.03 (71.06); H, 5.37(5.24); N, 12.34 (12.32).

#### 2.4.6. 4-((2-Hydroxyphenyl)(8-hydroxyquinolinyl)methylamino)-1,5-dimethyl-2-phenylpyrazol-3-one (**4f**)

Yield: 87%; IR (KBr, cm^−1^): 3422 (–OH), 3330 (–NH), 3021 (Ar–H), 2974 (–CH_3_), 2874 (–CH), 1695 (C=O), 1593 (C=N). ^1^H NMR (DMSO-d_6_, ppm): 2.54 (s, 3H, –CH_3_), 2.83 (s, 1H, –NH), 3.21 (s, 3H, –CH_3_), 4.55 (s, 1H, –OH), 5.31 (s, 1H, –OH), 5.43 (s, 1H, –CH), 6.72–7.12 (m, 5H, Ar), 7.14–7.20 (m, 4H, Ar), 7.25–8.22 (m, 5H, Ar). ^13^C NMR (DMSO-d_6_, ppm): 143.9 (C1′), 124.5 (C2′), 149.9 (C3′), 120.4 (C4′), 130.8 (C5′), 134.2 (C6′), 151.6 (C1′′), 126.9 (C2′′), 135.5 (C3′′), 128.1 (C4′′), 124.7 (C5′′), 129.6 (C6′′), 121.4 (C7′′), 147.2 (C8′′), 136.8 (C9′′), 45.5 (–CH), 128.7 (C1′′′), 134.6 (C2′′′), 161.5 (C3′′′), 117.3 (C4′′′), 33.0 (C5′′′), 133.6 (C6′′′), 121.7 (C7′′′), 127.4 (C8′′′), 120.8 (C9′′′), 127.7 (C10′′′), 120.9 (C11′′′). MS (C_27_H_24_N_4_O_3_): *m/z* 452 (M^+^, 100%). Elemental analysis: calcd. (found): C, 71.62 (71.66); H, 5.57(5.52); N, 12.12 (12.11).

#### 2.4.7. 4-((4–Chlorophenyl)(8-hydroxyquinolinyl)methylamino)-1,5-dimethyl-2-phenylpyrazol-3-one (**4g**)

Yield: 90%; IR (KBr, cm^−1^): 3427 (–OH), 3328 (–NH), 3018 (Ar–H), 2972 (–CH_3_), 2870 (–CH), 1692 (C=O), 1592 (C=N), 725 (C–Cl). ^1^H NMR (DMSO-d_6_, ppm): 2.51 (s, 3H, –CH_3_), 2.84 (s, 1H, –NH), 3.26 (s, 3H, –CH_3_), 5.35 (s, 1H, –OH), 5.45 (s, 1H, –CH), 6.73–6.88 (m, 5H, Ar), 7.00–7.15 (d, 4H, Ar), 7.23–8.21 (m, 5H, Ar). ^13^C NMR (DMSO-d_6_, ppm): 144.4 (C1′), 125.1 (C2′), 149.7 (C3′), 120.1 (C4′), 131.3 (C5′), 134.7 (C6′), 153.2 (C1′′), 126.4 (C2′′), 134.8 (C3′′), 127.3 (C4′′), 124.2 (C5′′), 128.5 (C6′′), 122.3 (C7′′), 147.3 (C8′′), 136.7 (C9′′), 52.1 (–CH), 128.6 (C1′′′), 135.5 (C2′′′), 161.8 (C3′′′), 116.9 (C4′′′), 33.4 (C5′′′), 132.7 (C6′′′), 121.2 (C7′′′), 127.8 (C8′′′), 121.7 (C9′′′), 128.2 (C10′′′), 121.5 (C11′′′). MS (C_27_H_23_ClN_4_O_2_): *m/z* 470 (M^+^, 100%). Elemental analysis: calcd. (found): C, 63.53 (63.56); H, 4.97(4.92); N, 13.90 (13.92).

#### 2.4.8. 4-((2–Chlorophenyl)(8-hydroxyquinolinyl)methylamino)-1,5-dimethyl-2-phenylpyrazol-3-one (**4h**)

Yield: 86%; IR (KBr, cm^−1^): 3425 (–OH), 3325 (–NH), 3022 (Ar–H), 2971 (–CH_3_), 2871 (–CH), 1690 (C=O), 1593 (C=N), 720 (C–Cl). ^1^H NMR (DMSO-d_6_, ppm): 2.53 (s, 3H, –CH_3_), 2.82 (s, 1H, –NH), 3.22 (s, 3H, –CH_3_), 5.33 (s, 1H, –OH), 5.42 (s, 1H, –CH), 6.71–7.11 (m, 5H, Ar), 7.18–7.22 (m, 4H, Ar), 7.26–8.23 (m, 5H, Ar). ^13^C NMR (DMSO-d_6_, ppm): 144.7 (C1′), 124.5 (C2′), 149.6 (C3′), 119.4 (C4′), 131.2 (C5′), 134.5 (C6′), 150.0 (C1′′), 125.8 (C2′′), 135.5 (C3′′), 127.5 (C4′′), 124.5 (C5′′), 129.2 (C6′′), 121.4 (C7′′), 148.2 (C8′′), 136.4 (C9′′), 47.5 (–CH), 128.9 (C1′′′), 135.6 (C2′′′), 161.6 (C3′′′), 116.4 (C4′′′), 33.8 (C5′′′), 132.2 (C6′′′), 120.6 (C7′′′), 127.7 (C8′′′), 121.3 (C9′′′), 128.5 (C10′′′), 121.2 (C11′′′). MS (C_27_H_23_ClN_4_O_2_): *m/z* 470 (M^+^, 100%). Elemental analysis: calcd. (found): C, 69.83 (69.86); H, 4.87(4.86); N, 12.90 (12.92).

## 3. Results and Discussion

### 3.1. Chemistry

In order to carry out the synthesis in a more efficient way that minimizes time and the amount of catalyst, a model reaction (Scheme 1) was magnetically stirred at room temperature using a naturally occurring mineral, fluorite, as a catalyst. The product was isolated by simple and usual workup with 92–95% of yield in simply 10–15 min. The catalyst was reused in at least eight reactions with no reduction in its efficiency. The structures of compounds **4**(**a–h**) were deduced from their spectral data. The solid state IR spectra of these compounds reveal a characteristic aromatic stretch between 3010 and 3022 cm^−1^. Sharp carbonyl (C=O) stretching vibrations for pyrazolone were seen around 1690–1695 cm^−1^. The presence of secondary amine N–H in the skeleton was confirmed from the stretching frequencies between 3325 and 3334 cm^−1^. The stretching vibrations for phenolic O–H were present between 3420 and 3428 cm^−1^. All other peaks in the spectra are in well agreement with the contents of functionalities in the synthesized molecules. The ^1^H NMR data of all compounds for the presence of aromatic protons reveal multiplets peak between 6.71 and 8.23 ppm. The spectra showed singlet around 5.41–5.45 ppm for –CH moiety in the compounds. The spectral data showed a characteristic singlet around 2.81–2.84 for the presence of N–H in the skeleton. Presence of singlet around 10.48–10.52 ppm reveals the presence of phenolic O–H on the ring. A singlet for three protons around 3.21–3.26 ppm indicates the presence of –CH_3_ on the ring. The ^13^C NMR spectrum of all the isolated compounds shows aliphatic –CH signals between 45.5 and 53.0 ppm. The other signals and peaks of ^1^H NMR, ^13^C NMR, and IR are in complete agreement with the assigned structures. The mass spectra of these compounds displayed a molecular ion peak at appropriate *m/z* values, which were corresponding well with the respected molecular formulas. All the compounds have given the satisfactory elemental analysis.

### 3.2. Investigation of *In Vivo* Anti-Inflammatory Activity

The screening results of compounds **4**(**a–h**) were summarized in Table 1. Oedema formation due to carrageenin in the rat paw is biphasic event. The initial phase is attributed to the release of histamine and serotonin. The second phase of oedema is due to the release of prostaglandins, protease, and lysosome. Subcutaneous injection of carrageenin into the rat paw produces inflammation resulting from plasma extravasations, increased tissue water, and plasma protein exudation along with neutrophil extravasations, all due to the metabolism of arachidonic acid. The first phase begins immediately after injection of carrageenin and diminishes in two hours. The second phase begins at the end of first phase and remains through third hour up to five hours.

In carrageenin administered animals the severe swelling was found to increase upto second hour and then started decreasing till fourth hour. The group treated by standard drug showed decreased paw oedema significantly throughout the period of study. The swelling was gradually reduced during the fourth hour in Diclofenac treated rats. The *in vivo* study reveals that compounds **4c**, **4d**, **4f**, and **4h **are significantly potent anti-inflammatory agents. It is noteworthy from Table 1 that all the synthesized compounds except **4a **and** 4b** were found to possess potential anti-inflammatory activity when compared with the reference drug.

### 3.3. Investigation of *In Vitro* Anthelmintic Activity

Anthelmintic activities of all prototypes were tested in this bioassay at various concentrations of 12.5, 25, 50, 100, and 150 mg/mL described in Table 2. All the investigational compounds **4**(**a–h**) acquired the anthelmintic activity at minimal dose of 12.5 mg/mL. Compounds **4a**, **4b**, **4e**, and** 4g** had shown their significant activity for time taken to paralysis and death when compared to the reference drug, Albendazole. Compounds **4d** and **4f** showed their moderate significant action for time taken to paralysis while compound **4a** exhibited their highly significant action for time taken to paralysis and death and which is almost equipotent action with respect to reference drug, Albendazole.

### 3.4. Molinspiration Calculations

All the synthesized compounds were screened theoretically by using online molinspiration software program [23]. Octanol-water partition coefficient (mi log P) calculation is used in rational drug design as a measure of molecular hydrophobicity which affects drug-receptor interaction. The polar surface area (PSA) of a molecule is the surface sum over all polar atoms, primarily oxygen and nitrogen including their attached hydrogens. A topological Polar Surface Area (TPSA) calculation is based on summation of tabulated surface contributions of polar fragments (i.e., bonding pattern) [24]. Lipophilicity (log *P* value) and polar surface area (PSA) values are the properties of the prediction of oral bioavailability of drug molecules [25, 26]. Therefore we have calculated these values for compounds **4(a–h)** and compared them with the values obtained for reference drugs, Diclofenac and Albendazole. Lipinski's rule of 5 is a thumb rule to evaluate drug-likeness (a chemical compound). The rule states that most drug-like molecules have log⁡*P* ≤ 5, molecular weight ≤ 500, number of hydrogen bond acceptors ≤ 10, and number of hydrogen bond donors ≤ 5. Molecules violating more than one of these rules may have problems with bioavailability [27]. For all the compounds, the calculated log *P* values are less than 5. The lowest degree of lipophilicity among all the compounds was exhibited by compounds **4a**, **4b**, **4e**, and **4f** which are an indication for good water solubility.  Table 3 shows that all the compounds are within this limit. All the synthesized compounds possess zero violation of the rule of 5.

## 4. Conclusion

In conclusion, a rapid and efficient synthesis of 4-aminoantipyrine derivatives via Betti reaction has been achieved. Higher yields were obtained in a less reaction time following a simple and usual workup. Fluorite is an efficient, reusable, benign, and cost-effective catalyst. The *in vivo *and* in vitro* screening results revealed that these derivatives possess potential anti-inflammatory and anthelmintic activity, respectively. Also the molinspiration calculations justify that the derivatives do not violate Lipinski's rule of 5; hence, a favourable bioavailability based on drug likeness is indicated.

## Figures and Tables

**Table 1:** Results of anti-inflammatory activity of 4-aminoantipyrine derivatives **4**(**a–h**).

Test Compds	Increase in paw volume at different time interval (h)^a^					% inhibition
	150 mg/kg					
	0	1	2	3	4
Control	0.32 ± 0.03	0.58 ± 0.02	0.83 ± 0.05	1.96 ± 0.01*	2.15 ± 0.04	—
**4a**	0.33 ± 0.02*	0.59 ± 0.01	0.80 ± 0.05*	0.78 ± 0.03	0.74 ± 0.01	47.27
**4b**	0.33 ± 0.05	0.56 ± 0.02	0.78 ± 0.03	0.77 ± 0.03	0.71 ± 0.03*	49.10
**4c**	0.30 ± 0.04*	0.47 ± 0.03	0.49 ± 0.02	0.34 ± 0.05*	0.33 ± 0.02	78.91
**4d**	0.31 ± 0.02*	0.47 ± 0.04	0.45 ± 0.01	0.37 ± 0.02	0.31 ± 0.03	74.82
**4e**	0.31 ± 0.05	0.42 ± 0.01*	0.46 ± 0.03	0.34 ± 0.02	0.32 ± 0.03*	72.73
**4f**	0.30 ± 0.04*	0.36 ± 0.04	0.42 ± 0.05	0.33 ± 0.01*	0.31 ± 0.04	74.55
**4g**	0.32 ± 0.05	0.54 ± 0.03*	0.66 ± 0.02*	0.49 ± 0.04	0.44 ± 0.01*	61.82
**4h**	0.30 ± 0.03	0.34 ± 0.01*	0.41 ± 0.05*	0.33 ± 0.04	0.31 ± 0.05*	76.45
Diclofenac (10 mg/kg)	0.30 ± 0.01	0.31 ± 0.03	0.33 ± 0.05*	0.12 ± 0.04	0.10 ± 0.02*	80.45

*Significantly different from control at *P* < 0.05.

^a^Results are expressed as mean ± SEM.

**Table 2:** Results of anthelmintic activity of 4-aminoantipyrine derivatives **4**(**a–h**).

Test Compds	12.5 mg/mL		25 mg/mL		50 mg/mL		100 mg/mL		150 mg/mL	
	Time of paralysis (min.)	Time of death (min.)	Time of paralysis (min.)	Time of death (min.)	Time of paralysis (min.)	Time of death (min.)	Time of paralysis (min.)	Time of death (min.)	Time of paralysis (min.)	Time of death (min.)
Control	—	—	—	—	—	—	—	—	—	—
ALB	21 ± 0.6	22 ± 0.5	18 ± 0.4*	19 ± 0.6	15 ± 1.0	17 ± 0.8	13 ± 1.2	14 ± 0.6*	10 ± 0.5	10 ± 1.8*
**4a**	22 ± 1.4	23 ± 0.5	19 ± 1.5	20 ± 0.7*	16 ± 1.0	17 ± 0.6	14 ± 0.7*	15 ± 1.3*	11 ± 0.5	11 ± 1.0*
**4b**	23 ± 1.5	23 ± 1.3*	20 ± 2.1*	21 ± 2.3	18 ± 1.2	20 ± 1.0	15 ± 1.2*	16 ± 1.7	12 ± 0.5	12 ± 0.3
**4c**	30 ± 0.6	31 ± 0.5*	28 ± 1.3	29 ± 1.0*	26 ± 0.7*	27 ± 0.9	22 ± 0.5	23 ± 0.7	20 ± 1.2*	19 ± 0.7
**4d**	39 ± 0.9	42 ± 1.5	37 ± 1.4	40 ± 1.2	35 ± 1.3	35 ± 1.2	33 ± 0.6	34 ± 0.7	31 ± 0.8	32 ± 0.4
**4e**	24 ± 0.8*	24 ± 1.3	21 ± 1.3	22 ± 0.1*	17 ± 0.6	17 ± 1.6*	14 ± 0.7	15 ± 1.0	12 ± 0.8*	12 ± 0.2
**4f**	30 ± 1.0*	30 ± 0.6	29 ± 0.3	30 ± 1.0	27 ± 1.2	28 ± 0.6*	24 ± 0.5	25 ± 1.2	20 ± 1.5	21 ±1.2
**4g**	21 ± 0.4	23 ± 0.7	19 ± 0.3	19 ± 1.1	16 ± 1.0	17 ± 0.6	13 ± 1.7	14 ± 0.6*	12 ± 1.0	12 ± 1.2
**4h**	29 ± 0.3	30 ± 0.5	26 ± 1.3	27 ± 1.0	24 ± 0.2*	24 ± 0.6*	21 ± 0.7	22 ± 0.4	18 ± 1.0	19 ± 1.2*

Standard drug, Albendazole (ALB), was used at 10 mg/mL; “—” indicates absence of activity in 24 h of administration. *Significantly different from ALB at *P* < 0.05.

**Table 3:** Molinspiration calculation of 4-aminoantipyrine derivatives **4**(**a–h**).

Compounds	MW	mi log *P*	TPSA	OH-HN	N violation	Volume
**4a**	481	3.98	117	2	0	421
**4b**	481	3.96	117	2	0	421
**4c**	466	4.10	81	2	0	423
**4d**	479	4.12	75	2	0	444
**4e**	452	3.57	92	3	0	406
**4f**	452	3.99	92	3	0	406
**4g**	470	4.72	72	2	0	411
**4h**	470	4.68	72	2	0	411
Diclofenac	296	4.56	49	2	0	238
Albendazole	265	2.74	67	2	0	234

MW: molecular weight; TPSA: Topological polar surface area.

## References

[1] Ugi I, Werner B, Dömling A (2003). The chemistry of isocyanides, their multicomponent reactions and their libraries. *Molecules*.

[2] Mannich C, Krosche W (1912). Ueber ein Kondensationsprodukt aus Formaldehyd, Ammoniak und Antipyrin. *Archiv der Pharmazie*.

[3] Cardellicchio C, Ciccarella G, Naso F, Schingaro E, Scordari F (1998). The Betti base: absolute configuration and routes to a family of related chiral nonracemic bases. *Tetrahedron Asymmetry*.

[4] Lu J, Xu X, Wang C, He J, Hu Y, Hu H (2002). Synthesis of chiral ligands derived from the Betti base and their use in the enantioselective addition of diethylzinc to aromatic aldehydes. *Tetrahedron Letters*.

[5] Shaterian HR, Yarahmadi H, Ghashang M (2008). An efficient, simple and expedition synthesis of 1-amidoalkyl-2-naphthols as ‘drug like’ molecules for biological screening. *Bioorganic and Medicinal Chemistry Letters*.

[6] McGettigan P, Henry D (2006). Cardiovascular risk and inhibition of cyclooxygenase: a systematic review of the observational studies of selective and nonselective inhibitors of cyclooxygenase 2. *Journal of the American Medical Association*.

[7] Robert A (1976). Antisecretory, antiulcer, cytoprotective and diarrheogenic properties of prostaglandins. *Advances in Prostaglandin and Thromboxane Research*.

[8] Kurumbail RG, Stevens AM, Gierse JK (1996). Structural basis for selective inhibition of cyciooxygenase-2 by anti-inflammatory agents. *Nature*.

[9] Tapiero H, Nguyen Ba G, Couvreur P, Tew KD (2002). Polyunsaturated fatty acids (PUFA) and eicosanoids in human health and pathologies. *Biomedicine and Pharmacotherapy*.

[10] Dharmasiri MG, Jayakody JRAC, Galhena G, Liyanage SSP, Ratnasooriya WD (2003). Anti-inflammatory and analgesic activities of mature fresh leaves of Vitex negundo. *Journal of Ethnopharmacology*.

[11] Burdulene D, Palaima A, Stumbryavichyute Z (1999). Synthesis and antiinflammatory activity of 4-aminoantipyrine derivatives of succinamides. *Pharmaceutical Chemistry Journal*.

[12] Turan-Zitouni G, Sivaci M, Kiliç FS, Erol K (2001). Synthesis of some triazolyl-antipyrine derivatives and investigation of analgesic activity. *European Journal of Medicinal Chemistry*.

[13] Alama MS, Choib JH, Lee DU (2012). Synthesis of novel Schiff base analogues of 4-amino-1,5-dimethyl-2-phenylpyrazol-3-one and their evaluation for antioxidant and anti-inflammatory activity. *Bioorganic Medicinal Chemistry*.

[14] Himaja M, Rai K, Anish KV, Ramana MV, Karigar AA (2012). Synthesis and evaluation of anthelmintic and insecticidal activities of 4-amino-antipyrine derivatives of amino acids and peptides. *Journal of Pharmaceutical and Scientific Innovation*.

[15] Sigroha S, Narasimhan B, Kumar P (2012). Design, synthesis, antimicrobial, anticancer evaluation, and QSAR studies of 4-(substituted benzylidene-amino)-1,5-dimethyl-2-phenyl-1,2-dihydropyrazol-3-ones. *Medicinal Chemistry Research*.

[16] Vaghasiya YK, Nair R, Soni M, Baluja S, Chanda S (2004). Synthesis, structural determination and antibacterial activity of compounds derived from vanillin and 4-aminoantipyrine. *Journal of the Serbian Chemical Society*.

[17] Wada S, Suzuki H (2003). Calcite and fluorite as catalyst for the Knövenagel condensation of malononitrile and methyl cyanoacetate under solvent-free conditions. *Tetrahedron Letters*.

[18] Mohanram I, Meshram J (2013). Evaluation of in vivo anti-inflammatory and analgesic activities of novel derivatives of Ugi-4CR. *Mini-Reviews in Medicinal Chemistry*.

[19] Mohanram I, Meshram J, Shaikh A, Kandpal B (2013). Microwave-assisted one-pot synthesis of bioactive Ugi-4CR using fluorite as benign and heterogeneous catalyst. *Synthetic Communications*.

[20] Winter CA, Risley EA, Nuss GW (1962). Carrageenin-induced edema in hind paw of the rat as an assay for antiiflammatory drugs. *Proceedings of the Society for Experimental Biology and Medicine*.

[21] Bhatt KR, Mehra RK, Shrivastava PN (1977). A simple method for recording antiinflammatory effects on rat paw oedema. *Indian Journal of Physiology and Pharmacology*.

[22] Gbolade AA, Adeyemi AA (2008). Anthelmintic activities of three medicinal plants from Nigeria. *Fitoterapia*.

[23] http://www.molinspiration.com/.

[24] Ertl P, Rohde B, Selzer P (2000). Fast calculation of molecular polar surface area as a sum of fragment-based contributions and its application to the prediction of drug transport properties. *Journal of Medicinal Chemistry*.

[25] Chang LCW, Spanjersberg RF, Von Frijtag Drabbe Künzel JK (2004). 2,4,6-Trisubstituted pyrimidines as a new class of selective adenosine A1 receptor antagonists. *Journal of Medicinal Chemistry*.

[26] Clark DE (1999). Rapid calculation of polar molecular surface area and its application to the prediction of transport phenomena. 1. Prediction of intestinal absorption. *Journal of Pharmaceutical Sciences*.

[27] Lipinski CA, Lombardo F, Dominy BW, Feeney PJ (2001). Experimental and computational approaches to estimate solubility and permeability in drug discovery and development settings. *Advanced Drug Delivery Reviews*.

